# Comparison of Locoregional Recurrence with Mastectomy *vs.* Breast Conserving Surgery in Pregnancy Associated Breast Cancer (PABC)

**DOI:** 10.3390/cancers1010012

**Published:** 2009-12-04

**Authors:** Sushil Beriwal, Bunja Rungruang, Atilla Soran, Darcy Thull, Joseph L. Kelley, Rohit Bhargava, Chyongchiou J. Lin, Paniti Sukumvanich

**Affiliations:** 1 Department of Radiation Oncology, Magee-Womens Hospital of UPMC, 300 Halket Street, Pittsburgh, PA 15213, USA; E-Mail: lincj@upmc.edu (C.J.L.); 2 Department of Gynecologic Oncology, Magee-Womens Hospital of UPMC, 300 Halket Street, Pittsburgh, PA 15213, USA; E-Mails: rungruangby@upmc.edu (B.R.); jkelley@upmc.edu (J.L.K.); psukumvanich@upmc.edu (P.S.); 3 Department of Surgery, Magee-Womens Hospital of UPMC, 300 Halket Street, Pittsburgh, PA 15213, USA; E-Mail: asoran@upmc.edu (A.S.); 4 Division of Hematology Oncology, Magee-Womens Hospital of UPMC, 300 Halket Street, Pittsburgh, PA 15213, USA; E-Mail: dthull@upmc.edu (D.T.); 5 Department of Pathology, Magee-Womens Hospital of UPMC, 300 Halket Street, Pittsburgh, PA 15213, USA; E-Mail: rbhargava@upmc.edu (R.B.); 6 Department of Family Medicine, School of Medicine, University of Pittsburgh, 3518 Fifth Avenue, Pittsburgh, PA 15261, USA; E-Mail: cjlin@pitt.edu (C.J.L.)

**Keywords:** pregnancy, cancer, surgery, mastectomy, breast conserving surgery

## Abstract

We have compared outcomes, including the locoregional recurrence, between mastectomy and breast conserving therapy in PABC. Patients were divided into those who were treated with mastectomies (group 1) and those with breast conserving surgery (group 2). The groups were comparable except for lower mean age in group 2 and more patients with stage III disease and higher number of nodes positive in the group 1. Five-year actuarial LRR, distant metastases free survival and overall survival in group 1 *vs.* 2 were 10% *vs.* 37%, 73% *vs.* 81% and 57% *vs.* 59% respectively. The patients with PABC treated with breast conserving therapy, despite having lower stage disease, have a higher risk of local regional recurrence in comparison with those treated with mastectomy.

## 1. Introduction

Pregnancy associated breast cancer (PABC) is defined as breast cancer that is diagnosed during a pregnancy or within one year postpartum. Breast cancer is the second most common malignancy diagnosed during pregnancy, affecting one in 1,500 to one in 4,200 [1,2] pregnancies. Historically, pregnancy-associated breast cancer was thought to be aggressive with poor outcome but more recently the prognosis of PABC been shown to be similar to that of non pregnant women when matched for age and stage [3,4,5,6,7,8].

The decision regarding the use of locoregional therapy is very challenging for these patients [5]. For those who present in the late-second or early-third trimester, breast conserving surgery (BCT) followed by radiotherapy is feasible provided radiation can be initiated within 8–12 weeks of surgery or adjuvant chemotherapy. There is very limited data on outcome with BCT with PABC although it is presumed that it would be similar for age and stage matched control. The goal of this study is to compare locoregional recurrence rate (LRR) between mastectomy and BCT in patients with PABC.

## 2. Materials and Methods

Sixty consecutive patients with nonmetastatic breast cancer diagnosed either during pregnancy or up to one year postpartum treated from 1990–2005 at Magee-Womens Hospital were retrospectively analyzed. The data was obtained from the hospital tumor registry. All patients received definitive locoregional treatment with either mastectomy or BCT. Decisions about locoregional treatment strategy, chemotherapy, and hormonal therapy were based on clinical staging, physician discretion, and patient preference. All patients underwent surgical evaluation of the axilla either with axillary dissection or sentinel lymph node biopsy followed by axillary dissection in the case of one or more positive sentinel lymph nodes. Patients treated with BCT received external beam radiation prescribed to the entire breast to a median dose of 50 Gy in 25 fractions followed by a boost to the tumor bed for an additional 10–16 Gy. Patients treated with postmastectomy radiation were prescribed to the chest wall and undissected draining lymphatics for a median dose of 50 Gy in 25 fractions, followed by a boost to the chest wall scar of an additional 10 Gy. Chemotherapy was generally doxorubicin based and patients with positive lymph nodes generally were treated with taxane-based therapy after its introduction. No patient received trastuzumab based adjuvant therapy. Fifty-one patients had chemotherapy of whom 31 had it administered neoadjuvantly.

The more advance stage between the pathologic or clinical staging was used for analysis in cases where patients received neoadjuvant chemotherapy. Local regional recurrence was defined as ipsilateral local (breast or chest wall) or regional nodal recurrence (including axillary, supraclavicular, infraclavicular, or internal mammary nodal beds). Any other site of recurrence was recorded as distant metastasis (DM). All LRRs were considered events regardless of their relation to DM in time.

All data analyses were performed using SPSS version 17.0 (SPSS, Inc., Chicago IL). The differences between the variables in the two groups were evaluated using chi square, Student’s t test, or Fischer’s exact test. Actuarial rates of LRR, DM, and overall survival (OS) were calculated using the Kaplan-Meier statistic, and comparison between the two groups was calculated using log-rank test. All *p* value calculations were two sided, and only *p* ≤ 0.05 was considered statistically significant. Multivariate analysis was performed using forward stepwise Cox proportional hazards regression model.

## 3. Results

Patients were divided into those who were treated with mastectomy group and those with BCT (BCT group). The demographics and disease & treatment characteristics including age, race, grade, family history, timing of pregnancy, BRCA1/2 mutation status, number of positive nodes, number of nodes dissected, stage, estrogen receptor status, lymphovascular space invasion (LVSI), and chemotherapy are shown in Table 1, Table 2, Table 3 and Table 4. All these were comparable between the two groups except for lower mean age in BCT group and more patients with unknown family history, stage III disease and higher number of nodes positive in the mastectomy group. The mean tumor size at presentation was 3.32 cm (0.8–8 cm) in the mastectomy group and 2.04 (1–4 cm) in the BCT group (p = 0.008). All patients had negative margins of at least 2 mm or more.

**Table 1:** Patient and Tumor Characteristics for both groups.

	Mastectomy N = 33 Mean (SD) /N(%)	BCT N = 27 Mean (SD) /N(%)	P Value
**Age, years**			
Mean (SD)	35.61	33.15	0.04
Range	24–42	24–44	
**Race**			
Caucasian	31 (93.9)	26 (96.3)	0.65
Afro American	1 (3.1)	1 (3.7)	
Other	1 (3.1)	0 (0.0)	
**Pregnancy Timing**			
Antepartum	15 (45.5)	16 (59.3)	0.312
Post partum	18 (54.5)	11 (40.7)	
**Family History**			
Positive	15 (45.5)	15 (55.6)	0.031
Negative	11 (33.3)	12 (44.4)	
Unknown	7 (21.2)	0 (0.0)	

**Figure 1:**
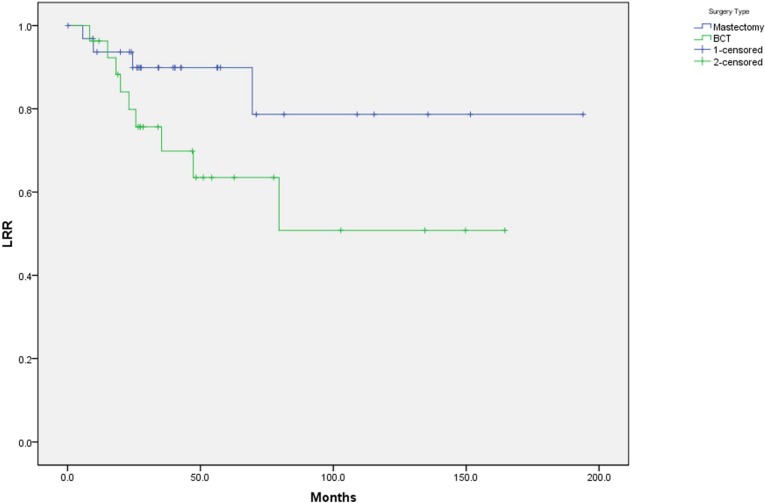
K-M Curve Showing Loco-regional Recurrence Free Survival.

All patients in the BCT group and 14 patients in the mastectomy group had adjuvant radiation therapy. The median time interval between surgery or last dose of adjuvant chemotherapy and adjuvant radiation therapy was four weeks (3–10 weeks). At mean follow up of 55 months (2–194 months), nine patients in BCT group and four patients in mastectomy group had local recurrence with five actuarial recurrence rate of 37% and 10% respectively (Figure 1; p = 0.04). Sites of recurrence for the BCT group included ipsilateral breast (seven patients) and axilla (two patients). Sites of recurrence for the mastectomy group include chest wall in four patients. On univariate analysis, the only factor significant for LRR was type of surgery with BCT having a higher recurrence rate than mastectomy. When excluding from analysis the two patients with nodal recurrence in the BCT group, the difference between the two groups was not statistically significant. On multivariate analysis including stage, nodal status, grade, age, LVSI, and type of surgery, no variable was associated with increased risk of recurrence.

Six patient in BCT group and eight in mastectomy group developed metastasis with five years, actuarial DM free rates of 81% and 71% (p = 0.68) respectively. On univariate analysis the presence of LRR did not increase the risk of distant metastases. The five years OS was 57% and 59% for the mastectomy and BCT group respectively (p = 0.85) (Table 5).

**Table 2:** Tumor Characteristics by Treatment Group.

	Mastectomy N = 33 N (%)	BCT N = 27 N (%)	P Value
**BRCA1/2 Mutation**			
Positive	3 (37.5)	4 (50.0)	0.7
Negative	5 (62.5)	4 (50.0)	
**ER**			
Negative	16 (48.5)	10 (37.0)	0.395
Positive	7 (21.2)	10 (37.0)	
Unknown	10 (30.3)	7 (25.9)	
**Nottingham Grade**			
1	0 (0.0)	1 (3.7)	0.05
2	1 (3.0)	7 (25.9)	
3	23 (69.7)	12 (44.4)	
Unknown	7 (21.2)	7 (25.9)	
**LVSI**			
Absent	13 (39.4)	13 (48.1)	0.720
Present	16 (48.5)	10 (37.0)	
Unknown	4 (12.1)	4 (14.8)	

**Table 3:** Treatment, Pathology, Status, and Staging by Treatment Group.

	Mastectomy N = 33 Mean (SD) /N(%)	BCT N = 27 Mean (SD) /N(%)	P Value
**Chemotherapy**			
No	7 (21.2)	2 (7.4)	0.166
Yes	26 (78.8)	25 (92.6)	
**Neo-Adjuvant Chemotherapy**			
Yes	13 (0.50)	18 (0.72)	0.08
No	13 (0.50)	7 (0.28)	
**Type of LN Surgery**			
ALND	23 (69.7)	19 (70.4)	0.967
SLND	8 (24.2)	6 (22.2)	
SLND plus ALND	2 (6.1)	2 (2.4)	

ALND = Axillary Lymph Node Dissection; SLND = Sentinel Lymph Node Dissection.

**Table 4:** Lymph Nodes Status, Stage, and Grade.

	Mastectomy N = 33	BCT N = 27	P Value
**Pathological LN Status**			
Positive	15 (51.7)	14 (51.9)	0.622
Negative	14 (48.3)	13 (48.1)	
**No. Nodes Dissected**			
Mean	12.0 (6.5)	15.3 (8.4)	0.099
Median	13	15	
Range	1–30	2–31	
**No. Nodes Positive**			
Mean	3.6 (5.5)	0 (1.8)	0.015
Median	1	0	
Range	0–17	0–9	
**T Stage**			
Tis	2 (6.1)	0 (0.0)	0.006
T1	6 (18.2)	13 (48.1)	
T2	11 (33.3)	14 (51.9)	
T3	8 (24.2)	0 (0.0)	
T4	3 (9.1)	0 (0.0)	
Tx	3 (9.1)	0 (0.0)	
**AJCC Stage**			
0	2 (6.1)	0 (0.0)	0.077
I	3 (9.1)	3 (11.1)	
IIA	10 (30.3)	15 (55.6)	
IIB	4 (12.1)	5 (18.5)	
IIIA	8 (24.2)	4 (14.8)	
IIIC	6 (18.2)	0 (0.0)	
**Stage Grouping**			
0	2 (6.1)	0 (0.0)	0.044
1	3 (9.1)	3 (11.1)	
2	17 (42.4)	22 (74.1)	
3	17 (42.4)	2 (14.8)	
**Follow up**			
Mean (Months)	51.3	61	0.401
Range	2–194	9.7–164	

SD: standard deviation.

**Table 5:** Five years actuarial rates of Loco-regional Recurrence, Distant Mets Free, Disease Free Survival and Overall Survival.

5 yrs (actuarial)	Group 1 % N = 33 (%)	Group 2 % N = 27 (%)	P value
Locoregional Recurrence	10	37	0.04
Distant Mets Free	73	81	0.68
Disease Free Survival	60	71	0.52
Overall Survival	57	59	0.85

## 4. Conclusions

The management of PABC is very challenging for the treating physicians. Clinical decisions are hampered by lack of prospective clinical studies and long-term outcome data [5]. The optimal locoregional therapy is not well defined but is presumed that BCT should have similar outcome as nonpregnant women although the pregnant breast with large anastomosing network of ducts and sizeable blood/lymph vessels is considered to be anatomically different from the nonpregnant breast. There are very limited studies with BCT for PABC in the literature [8,9,10]. In one small study with nine patients treated with BCT with median follow up of 24 months, there was no local recurrence and three distant metastases [10]. However, it should be noted that the median tumor size in this series was 1.5 cm which is smaller than typical for PABC. Similar findings were reported by a group from MD Anderson where four patients were treated with neoadjuvant chemotherapy followed by BCT [9]. In this small series with median follow up of 44 months, none of the four patients had local recurrence. In contrast, our study with a larger number of patients suggests that the LRR is higher in patients treated with BCT in comparison to mastectomy even though mastectomy group had more patients with advanced stage disease. Our results were not significant on multivariate analysis probably because of the small sample size.

The young age of patients with PABC may be the reason why a higher LRR rate is seen in patients treated with BCT. The mean age in the BCT group was 32 years in our series. Clinical studies have suggested that young patients with breast cancer consistently have worse outcomes than those who develop the disease later in life [11,12,13,14,15,16,17]. Many series found that patients 45 years or younger consistently have higher LRR rates when treated with BCT and some have reported that this translates into decreased overall survival [1,16,18]. Elkhuizen, *et al.* reported a 28.0% rate of ten-year LRR for patients 35 years or younger [18]. Similarly in an analysis of the European Organization for Research and Treatment of Cancer (EORTC) trials of patients with Stage I and II breast cancers suggested that both young age and BCT are independent risk factors for LRR [19]. In a recent retrospective study of young patients (≤35 years) for patients with Stage II disease, the best locoregional control rates were achieved with mastectomy plus radiation in comparison with mastectomy alone or BCT [20]. However, for patients with Stage I disease, similar outcomes were achieved with BCT and mastectomy. The LRR in our series with BCT was even higher probably because of advance stage of disease associated with PABC in comparison to these series in nonpregnant women. Some of the treatment interventions being evaluated in younger patients including MRI for assessment of additional disease and higher boost dose may also help in improving outcome PABC patients treated with BCT.

The rationale for the higher recurrence seen in young patients has been hypothesized to be biologically aggressive disease characterized by features including higher grade, LVSI, extensive intraductal component, and ER negativity [21]. These histopathological features are similar to what have been reported in PABC [5,22]. Now gene expression analysis has identified several breast cancer subtypes, including basal-like, human epidermal growth factor receptor-2 positive/estrogen receptor negative (HER2+/ER-), luminal A, and luminal B which predicts for outcome and response [23]. In our ongoing study we are applying immunohistochemical surrogates for subtyping PABC to see if the aggressive type is more common in PABC and also comparing it to age matched control.

This current series is the largest study in literature on PABC evaluating locoregional recurrence differences based on type of surgery. This difference in LRR observed in our study is not definitive but is certainly hypothesis generating. There are several limitations to our investigation including the retrospective nature and heterogeneity of treatment over the decade long time period for the study. There are also inherent and potentially hidden biases that may have governed treatment decisions and may impact on outcomes.

Due to the relative rarity of PABC, large prospective studies are not feasible. There is a need for multicenter cooperation and a central registry that will collect and follow a large number of cases of PABC. This will facilitate better understanding of the biology of the disease and help to optimize management strategy including loco regional therapy.
